# Just data? Solidarity and justice in data-driven medicine

**DOI:** 10.1186/s40504-020-00101-7

**Published:** 2020-08-25

**Authors:** Patrik Hummel, Matthias Braun

**Affiliations:** Department of Theology, Friedrich-Alexander University Erlangen-Nürnberg (FAU), Kochstraße 6, 91054 Erlangen, Germany

**Keywords:** Data-driven medicine, Precision medicine, Algorithm ethics, Data ethics, Justice, Solidarity, Controllability, Privacy

## Abstract

This paper argues that data-driven medicine gives rise to a particular normative challenge. Against the backdrop of a distinction between the *good* and the *right*, harnessing personal health data towards the development and refinement of data-driven medicine is to be welcomed from the perspective of the *good*. Enacting solidarity drives progress in research and clinical practice. At the same time, such acts of sharing could—especially considering current developments in big data and artificial intelligence—compromise the *right* by leading to injustices and affecting concrete modes of individual self-determination. In order to address this potential tension, two key elements for ethical reflection on data-driven medicine are proposed: the *controllability* of information flows, including technical infrastructures that are conducive towards controllability, and a paradigm shift towards *output-orientation* in governance and policy.

## Introduction

Digitization and datafication shape and transform health research and clinical practice. These transformations yield new opportunities and insights, but also pose challenges to individuals and society. One example is the use of COVID-19 tracing apps that alert users if they have been in contact with someone carrying the coronavirus SARS-CoV-2, and for example suggest self-quarantine on this basis. Tracing apps are part of a bundle of measures to tackle the COVID-19 pandemic.

One motivation for people to install and use such an app may be that they want to contribute their part to a common good, to show solidarity, and to donate their data for public health surveillance and research. Indeed, the sharing of data is essential for digital contact tracing and data-driven health applications in general to function effectively and yield sound results. Such technologies, however, can come at a price. Collecting and using (sensitive) personal data may in principle attain benefits and strengthen freedom, e.g., if lockdowns are prevented by early detection and interruption of transmission chains. However, it can also compromise privacy, social equality, and fairness, e.g., when access to a tracing app presupposes access to economic or technological resources, or when refusal, traced infection or immunity status constrain modes of freedom and inclusion for some or even most individuals.

As these observations illustrate, there is a need to reflect on the ethical aspects of data-driven health. In the following, we contend that such an ethical reflection—not only on tracing apps, but also many other digital health applications—would need to address at least two topics: our pursuits of the *good*, of which acts of solidarity are paradigmatic instances, and the *right*, of which justice is one central component. Our objective is to examine the interplay between both spheres and the significance of this distinction for an ethics of data-driven medicine.

With data-driven medicine (Shah and Tenenbaum 2012; Torkamani et al. 2017), we refer to medical research and care that rests upon consideration of large amounts of data and deploys algorithmic tools to guide prediction, prevention, diagnosis, and treatment, for example in attempts to steer towards *precision* medicine (Ashley 2015; Collins and Varmus 2015; Hawgood et al. 2015) that promises to tailor prevention, diagnostics, and treatments to the specific characteristics and circumstances of individual patients. With this understanding of data-driven medicine at hand, we draw on a distinction between the *good* as denoting what constitutes intrinsic value and/or contributes to a fulfilled life, and the *right* as the domain of impartial standards of justice. Our claim is that sharing one’s health data can promote the *good*, but threatens to undercut aspects of the *right*. Specifically, we discuss three examples: the inequitable consideration of data from different sources and populations, and algorithmic injustice reflecting and amplifying these inequities; shortcomings in distributing access to data-driven medicine and addressing risk profiles uncovered by its predictive methods; and incentivization and disciplining effects of continuous tracking of health data. Our positive proposal is that we stand a chance to seize the potentials of data-driven medicine and their value towards the *good* while being mindful of constraints from the sphere of the *right* if we focus on two goals. First, data flows shall be *controllable* by the individuals whose data is being processed, which from our perspective involves the right to know and to affect which kind of person-related inferences can be drawn on the basis of their data. In order to get there, technical infrastructures must be aligned with the ideal of controllability. Second, governance and policy would have to broaden their focus in order to proceed from an input- towards an *output-orientation* that addresses the downstream effects of data-intensive tools on the opportunities of individuals to exercise self-determination and to partake in societal endeavors.

## Sharing data for the sake of the good

So far, debates on ethical aspects of data-driven medicine focus primarily on identifying and spelling out relevant principles of biomedical ethics in contexts of intensified datafication and computerized clinical decision support. However, there are actually a number of related but distinct pursuits that one might subsume under the heading of ‘ethics’—distinctions which have not yet been made explicit when reflecting on data-driven medicine.

As one example, consider Ross (1930), who in the course of advancing his realist, non-naturalist intuitionism about morality, distinguishes two domains of moral theorizing: the *right* and the *good*. Reflection on common-sense morality suggests that both spheres are indefinable, and in particular cannot be reduced to each other. Moreover, he puts forward a pluralist ethics which countenances not one, but a whole set of irreducible principles at the levels of the *right* and the *good*, respectively. This and his understanding of a *prima facie* duty have been influential for *principlism* (Beauchamp and Childress 2013, 15–16). In Ross’s terminology, *rightness* is a feature of *acts*, and rests on duties to fidelity, reparation, gratitude, promotion of a maximum of aggregate good, and non-maleficence (Ross 1930, 21). *Goodness* denotes intrinsic value, and is a feature of states of affairs, motives, and outcomes. Specifically, he lists virtue, pleasure, knowledge, and justice (Ross 1930, 134–41).

As another example, consider Habermas (1993), who draws a distinction between *pragmatic*, *ethical*, and *moral* employments of practical reason. In pragmatic employments, the will is fixed, and the focus is on techniques and strategies for implementation. In contrast, some decisions require reflection upon the will itself and what it takes to lead a good life: who we are, who we would like to become, and how we would like to live. In Habermas’ terminology, they pertain to the sphere of *ethics*. Yet another class of decisions transcends the perspective of the particular agent. The interests of others are taken into consideration impartially. For Habermas, a maxim is “*unjust* if its general observance is not equally good for all” (Habermas 1993, 7). Examining maxims in these ways is a matter of *moral* deliberation. It does not concern what is good for a particular agent, but raises the question of whether “a generally observed maxim is suitable to regulate our communal existence […], whether I can will that a maxim should be followed by everyone as a general law” (ibid.).

In the following, we seek to harness these framings while sidestepping the following issues as far as possible. First, we remain agnostic on the exact relation between the *right* and the *good*. For example, as can be seen from these brief expositions, Habermas seems to assume *some* interdependence between the *good* and the *right*, whereas Ross emphasizes their distinctness. What matters for our purposes is that both spheres can be distinguished and do not coincide. Second, we sidestep the question of how Ross’ and Habermas’ framings relate to each other, for example to what extent there is overlap between their notions of the *good*, and specifically to which sphere justice ultimately pertains. Ross acknowledges that the status of justice is complicated (Ross 1930, 26–27), but ultimately locates it, as mentioned, in the sphere of the *good*. In contrast, Habermas seems to locate justice in the sphere of the *right*, and we will accord with this in the following.

Setting these issues aside, let us note that extant debates on the ethical challenges in data-driven medicine leave open which of the foregoing spheres they address, i.e., whether *rightness* or *goodness* is at stake, and/or whether *pragmatic*, *ethical*, or *moral* considerations are called for. One initial proposal of ours is that in the context of data-driven medicine, the sharing of health data is in principle to be welcomed from the perspective of the *good*, i.e., bears the potential to realize intrinsic value, to enable fulfilled lives, and to put individuals in a position to promote the well-being of others.

As one example, availability of data opens up new avenues in biomedical research, public health, and clinical care by improving evidence bases, facilitating sound decision-making, and optimizing health outcomes. Computerized, algorithmic, and machine learning tools such as neural networks can be set up to recognize patterns in patient data, historical case data, chosen treatments, and outcomes, thereby highlighting patterns that would have otherwise escaped our attention. They can also be used as a safety mechanism to check proposed treatments for consistency with past choices. Independently of how such tools are put to work, sufficient amounts of data from the patient *and* her peers are needed to arrive at suitable reference classes, to train algorithmic applications, and to attune them to the envisioned task. For these reasons, generating data and making them accessible can in principle be welcomed from the perspective of the *good*. In Ross’ terms, we can note that such availability paves the way for knowledge generation and the optimized treatment of health conditions that inhibit pleasure. In Habermas’ terms, we can note that data-driven biomedical innovation can facilitate pursuits of our own notion of a fulfilled life.

Moreover, the needed data result from a bottom-up process of individuals sharing their personal data, ideally culminating in knowledge bases that strengthen the overall health of a population. In this sense, data-driven medicine, despite the individualistic spirit that underlies the idea of tailoring interventions to specific patients, is a context in which individual and collective good are *intertwined* (Sharon 2017, 100). The decision of individuals to share personal health data can be driven by a range of different motivations. On the one end of the spectrum, reasons of self-interest are conceivable. Such motivations are likely especially in settings where the prospect of benefitting from innovative tools is directly conditional upon contributing one’s data. The individual hopes to gain a better understanding of herself, her body, health, diseases, and risks of disease. Ideally, such knowledge can inform the choice of health services she receives and enhance their effectiveness.

On the other hand, sharing one’s data can be the result of motivations that do not rest on individualistic or particularistic goals, such as improvements of one’s own health. Sometimes, agents seek to transcend their individual sphere. Contributing to processes of scientific discovery and innovation by sharing one’s health data is one way to reinforce and to invest in the broader social context in which one exercises self-determination. It is not just that data sharing can be altruistic and beneficial. It can also be a way to participate in the pursuit of collective goals, and to play a part in constituting and reinforcing community. Indeed, gift theorists highlight that certain acts of giving are only fully understood if their *aneconomic* (Derrida 1992) aspects are taken into account. Gifts involve endowments, are not intended to prompt a return, do not merely advance self-interest, and convey symbolic, non-commodifiable aspects through recognition, dedication, and an investment of the donor *herself* into what is being given (Hénaff 2010, 2013). Such acts exceed a logic of exchange because they neither aim at a return nor could be offset against one. Gift theories make us aware that acts of giving can generate and reinforce social bonds, and open up new options in social space (Dabrock 2015, Braun 2017)*.*

Specifically, acts of giving can be driven by solidarity. Prainsack and Buyx define solidarity as signifying “shared practices reflecting a collective commitment to carry ‘costs’ (financial, social, emotional, or otherwise) to assist others” (Prainsack and Buyx 2012, 346, italics removed). Unlike altruism and charity, solidarity is based on the agent “recogniz[ing] sameness or similarity in at least one relevant respect” (ibid., italics removed). In subsequent work, they argue that if an individual knowingly contributes data to a database that aims to create social value (as opposed to pursuing private sector interests), and accepts the costs of her act of sharing without the expectation to be rewarded, then her contribution is plausibly framed as an act of solidarity. Institutions that are transparent about how they use data, seek to generate social value in the first place, and do not offer inappropriate compensation to data sharing individuals are implementing solidarity-based governance (Prainsack and Buyx 2017, chap. 5).

Solidarity and justice share certain features, but also differ in crucial respects. In some sense, both concern the (re-)allocation of goods, but they differ with regards to the grounds that motivate these allocations. Justice alludes to fundamental and universal standards of what is owed to individuals, in a sense to be specified by one’s preferred substantive theory of justice. It is universal, gives rise to an unconditional *ought*, but does not require going beyond these foundational standards. Solidarity pertains to the (re-)distribution of goods that exceeds these minimal, universal standards. The degree to which we have reason to enact solidarity varies. We do not choose to enact the latter as a result of mandatoriness or even coercion, but because of further motivations not already implicated by justice. As indicated by Prainsack and Buyx, solidarity motivates this distribution by reference to distinctive forms of relatedness amongst individuals. Dabrock (2012, chap. 5) proposes to conceive of these social ties on the model of Ricœur’s (2005) gift-theoretic remarks on giving and gratitude: they are non-calculatory by virtue of not aiming at a return from the recipient, but resting upon a non-instrumental interest in fostering community. Ideally, this gives rise to gratitude in the recipient, who is then free to give back. Rather than “annul[ling] the gift” (Derrida 1992, 13) by introducing aspects of compensation, successive acts of giving can each retain an asymmetry between giver and recipient. Iterations of such acts and their recognition can deepen modes of integration amongst individuals. A sense of mutual obligation results, but one that has its roots in something exceptional rather than the universal, foundational norms of justice. In this way, acts of solidarity occupy a middle ground between justice and love, charity, or beneficence: not driven by a categorical *ought*, but also not just something extra, unowed to the non-intimate other.

Qualitative empirical evidence suggests that attitudes of solidarity and/or altruism might play a role in data-sharing behavior. For example, Facio and colleagues suggest that individuals who contribute their whole-genome sequencing data to research intend to help others, e.g., those at risk of genetic disorders, and to contribute towards genetics or health, “[b]ecause I can give back and be part of groundbreaking (and potentially life saving) research” (Facio et al. 2011, 1214). Similarly, Oliver et al. (2012) queried subjects about expected benefits of sharing their genomic data. Participants were anticipating to help future patients, in particular those with a similar condition, to advance medical knowledge, and contribute to general societal benefits realized by genomic research. In both antecedently reported data sharing preferences and actual data sharing decisions throughout the study, individuals showed a tendency to prioritize these benefits over individual privacy concerns. Mählmann et al. report that a majority of individuals in their sample were willing to share their genomic data, and that they were “driven by altruistic motivations of wanting to contribute to the greater good and accelerating research to improve the health of society” (Mählmann et al. 2017). Some individuals even share their genomic data without reference to a specific research project or purpose. Haeusermann et al. (2017) examine the attitudes of contributors to OpenSNP, a freely accessible database of genomic data published under a Creative Commons Zero license. While some of their motivations are primarily self-regarding, such as curiosity and the desire to learn more about their genome, other motivations indicate a willingness to contribute towards a common good, such as the desire to advance medical research and to improve genetic testing. A reoccurring theme throughout these studies is that while self-interested reasons certainly play a role in explanations of acts of sharing one’s personal health data, at least some of the motivational drivers go further.

There is room for additional research, for example on the following three questions. First, how pervasive are these other-regarding motivational drivers? For example, are they dominant across all populations, or do some demographics stand out? Second, a more fine-grained stratification of examined motivational drivers would be desirable. For example, to what extent are other-regarding motivations of data sharing indicative of—in Prainsack and Buyx’ terminology—solidarity rather than charity? That is, in what sense and to what degree do they rest upon recognition of reciprocity, sameness, and symmetry with potential beneficiaries of the envisioned research (Braun 2017)? Third, which safeguards and control mechanisms do individuals expect in order to facilitate exercises of other-regarding dispositions to share data? From the perspective of these individuals, what are desired and appropriate governance mechanisms?

For our purposes, we can highlight these questions as interesting, but largely sidestep the details of answering them. We do not claim that individual attitudes cohere with pictures of solidarity and gift-giving in the sense outlined above uniformly, consistently, and with statistical significance. We propose these pictures not as empirical claims about the motivations and attitudes of a majority of individuals, but as descriptive schemes to capture a set of target phenomena in all its complexity. Our claim is not that we must employ the descriptive schemes of gift-giving and solidarity when framing decisions to share personal health data, just that these schemes highlight attitudes, motivations, and intentions that might have otherwise escaped our attention. Neither do we claim that individuals *must* give, and that the importance of privacy should be deflated. Our observations are compatible with leaving it wholly up to the individual to reflect upon whether and how she seeks to contribute to the good of others, and if desired to withhold rather than to share data. The idea that sharing data is in principle to be welcomed from the perspective of the *good* does not preempt the question of whether data should be shared in a given case.

We have argued that an ethics of data-driven medicine should consider and make explicit whether and when it addresses the *right* versus the *good*, and how the sharing of data in the context of data-driven medicine can advance the *good*. Individuals’ pursuits of their notions of the good life sometimes take the form of giving and gifting. Data is a valuable resource that individuals can give, and sharing them can be intended as an act of solidarity and an attempt to contribute to a greater good.

## Data sharing at the expense of the right

While we have just argued that the availability of health data for the purposes of data-driven medicine can in principle be welcomed from the perspective of the *good*, we now turn to potential consequences for the domain of the *right*, which we illustrate by reference to three specific examples. If the sharing of data is essential to data-driven medicine, and the sharing of data renders individuals more prone to encounter injustices, then questions of justice are likely to be raised and elevated when developing and applying methods of data-driven medicine.

### Algorithmic injustice

Datafication and increasing reliance on algorithms shape decision-making processes across societal subsystems. The digital becomes linked with the analogue, merging them into one unified *onlife* (Floridi 2015). Advances in computing power, especially through automatized, algorithmic processing, provide new ways to sample, analyze, and refigure these large amounts of data. These technologies permeate social interactions, marketing, search engines, finance, mobility, recruitment processes, juridical proceedings, clinical decision-making, etc. Across these contexts, some believe (Pasquale 2015)—whereas others dispute (Kroll 2018)—that decision-making relies on algorithms that are insufficiently transparent (Burrell 2016) to designers, users, and those affected. If users cannot always trace how outputs come about, and which underlying phenomena and mechanisms they track, yet at the same time AI-driven decision-making becomes ubiquitous, one might worry that meaningful human control is ceded rather than enhanced. In data-driven medicine advanced by various algorithmic tools, research and care can sometimes proceed on the basis of considering large amounts of data and providing algorithm-driven classifications *without* a thorough understanding of the underlying mechanisms of the model (Zhu and Zheng 2018). Whether this introduces distinctive complications for explainability, or whether these challenges resemble those of statistical modelling approaches, e.g., confounding that complicates hypotheses, is up for debate.

Either way, once research impacts practice, whether in biomedicine or beyond, opacity is significant especially because datafication and algorithmic tools not only set innovation and value generation in motion, but can also render data subjects susceptible to disadvantages, asymmetries, and exploitations. It is because of challenges like these that authors call for systematic consideration of the ethical issues in the development and application of algorithms. For example, Mittelstadt et al. describe problem areas and concepts “jointly sufficient for a principled organisation of the field” (Mittelstadt et al. 2016, 2): epistemic concerns about the quality of evidence provided by algorithms, unfair outcomes brought about by algorithms, transformative effects, i.e., reconceptualizations of worldviews and reconfigurations of social and political organization, and a lack of traceability inhibiting debugging and appropriate distributions of responsibility for harms caused by algorithms. Other framings of the ethics of algorithms differ slightly in their emphases. For example, Zarsky (2015) and Danaher et al. (2017) each distinguish two main key challenges of algorithmic governance: while Zarsky focuses on efficiency and fairness, Danaher and colleagues highlight effectiveness and legitimacy as the central issues. In the following, we take our cue from authors who specifically refer to the notion of *justice* and argue that it should play a prominent role in designing, deploying, and governing such technologies. They caution against automating inequality (Eubanks 2018), algorithms of oppression (Noble 2018), and call for data justice (Taylor 2017; Dencik et al. 2018) for individuals, but also groups and populations (Mittelstadt 2017; Taylor et al. 2017) as a guide across all stages of designing and refining algorithms.

One cause of injustices and their perpetuation by data-driven processes is that the latter can fail to consider data from different sources and populations. As a result, the characteristics of particular individuals or groups are over- or underrepresented. This can lead to inequitable reflection in the decision-making rules of the algorithmic tool. Consider the use of machine learning applications in dermatology where convolutional neural networks have been shown to perform on par with (Esteva et al. 2017) or even better than (Haenssle et al. 2018) dermatologists in classifying images of skin lesions and distinguishing benign and malignant moles. Adamson and Smith (2018) highlight that like any machine learning application, these networks require data for training, testing, and fine-tuning. However, as of now, a disproportionate amount of patient data used to learn such applications comes from fair-skinned populations. “Thus, no matter how advanced the algorithm, it may underperform on images of lesions in skin of color” (Adamson and Smith 2018). This worry generalizes to other variables of diseases, such as lesion location, patient age, and degree of sun damage: if the data contain a bias along these dimensions, some classes of patients will be left behind. If such issues materialize on a broader scale, it is conceivable that inequities in the allocation of benefits translate into structural injustices where certain classes of individuals are systematically disadvantaged in terms of how well the healthcare system caters to their needs. Pre-existing health disparities could get perpetuated, reinforced and exacerbated.

Of course, current algorithmic applications in the clinic are not plausibly regarded as autonomous decision-making entities. They are built up from human knowledge, for example when humans annotate data based on which the system learns. And they also do not make decisions themselves. At least until we encounter AI-driven clinical applications that are *fully automated* (in the sense of Yu et al. 2018), algorithmic tools in the clinic will merely provide recommendations to an expert, who then takes such outputs into consideration, plausibly in a *shared* decision-making (Elwyn et al. 2012) process with the patient. In this context, the possibility of bias could be flagged to the decision-makers. This, however, will only address a part of the problem. First, it presupposes that decision-makers are in a position to discount AI-driven evidence for potential system bias, which can be challenging epistemically. Second, even then, there will still be use cases where flagging bias will be insufficient to align service quality. For example, consider a machine learning application that is used as a safeguard to flag malignant moles that were overlooked during screening. If the training data was lopsided as in the Adamson and Smith scenario, skin color could affect the patient’s chances of benefitting from this AI-driven safeguard.

### Uncovering risk profiles

Second, data-intensive clinical applications are likely to eventually lead to significant increases in predictive power. Not always will predictions be straightforward. For example, based on an interview study with parents and clinicians involved in newborn screening, Timmermans and Buchbinder (2010) coin the concept *patients-in-waiting* to refer to individuals with screening values beyond normalcy, but who due to ambiguity and uncertainty in and around screening methods and results are unable to receive a clear-cut prognosis about their condition and progression timeline. But generally, the hope is that with data-driven research and development, knowledge of genetic and genomic risk factors, biomarkers, and dispositions will improve, both on the level of biomedical research where relationships between biomarkers and risks of disease are being examined, and at the level of risk profiles of individual patients in the clinic. Due to polygenetic and multifactorial etiologies together with the importance of environmental, behavioral, and nutritional parameters, such relationships are typically probabilistic. Care must be taken to ensure that individuals properly understand the content and limitations of their data. Knowledge of these relationships then invites a shift from a curative paradigm aiming to repair dysfunctions towards the inclusion of prevention-oriented interventions aiming to raise awareness and to enable decision-making before pathologies materialize.

There is a question whether advances in prediction and prevention will benefit those individuals who could enjoy the highest benefits from improved prediction. Instead, they might primarily help those who already enjoy favorable determinants of health in the first place and thus would have been less likely to progress to disease than members from other parts of society (Dabrock 2016, 291–92). Similar challenges arise at the international level. For example, with regards to applications of data-intensive genomics to clinical and public health decision-making on infectious diseases, we might worry that “[t]he majority of research investment comes from high-income countries, whereas the highest burden of infectious disease is in the developing world. The kind of research likely to have the greatest global benefits might not be given funding priority by countries with the greatest resources” (Geller et al. 2014, 8).

Increased predictive power can bring previously unknown risks to light. One interesting class of individuals is the *healthy* *ill* (Hubbard 1993): individuals who are currently symptom-free, but whose risk profile suggests that they are likely to progress to disease in the future. The availability and awareness of personal risk profiles alone can lead individuals to reevaluate their subjective level of health. For example, prompted by the actress Angelina Jolie who decided to undergo mastectomy upon genetic testing for the *BRCA1* gene, there was a surge in referrals related to breast cancer family history, demand for *BRCA1/2* testing, and enquiries about mastectomy—subsequently described as the Angelina Jolie effect (Evans et al. 2014).

Increased transparency of risk profiles opens up the possibility that injustices with regards to *management* of these risks arise, for example when the health system fails to treat risk profiles of comparable severity on par. Meier et al. (2017a) propose to distinguish the *healthy* *sick* from the *healthy* *ill*: the former class comprises those symptom-free patients whose risk of progressing to disease is recognized by society as meeting a threshold that warrants preventive interventions, in the sense that the public health system carries some or all of the costs of preventive steps. In further work, the authors argue that so far, the German health system fails to treat *healthy* *ills* on par (Meier et al. 2017b, 2018). For example, even before progressing to disease, Factor V Leiden carriers are entitled to heparin or phenprocoumon. But despite also bearing significant risk of disease, no analogous preventive measures are being provided in the case of *BRCA1/2.* One question of justice is how seemingly similar risk profiles that have become apparent through increased datafication and data-intensive processing can differ in entitlements to access public health services. This question involves a *distributive* aspect insofar as it concerns the fair allocation of resources (Marckmann 2016, 141), but also has a *procedural* dimension insofar as it demands transparency about the priorities and principles that guide the relevant decision-making processes.

### Ubiquitous datafication and tracking

Third, data from health apps, wearables, sensors, and other self-tracking practices can be the basis for increased awareness and improvements of health, but can also raise questions of justice. To begin with, knowingly or not such data tend to be shared with the service provider and third parties, which can result in undue downstream effects on the opportunities data subjects enjoy in social space. Moreover, pervasive datafication can shape and reinforce incentivization and sanctioning structures, such as more or less overt imperatives to participate in the tracking practice itself. For example, the effectiveness of tracing apps for COVID-19 is dependent on a sufficient number of individuals downloading and deploying such apps. The potential public health value of such endeavors can result in implicit deflations of privacy concerns, and tacit expectations that in view of the potential benefits for public health, individuals *should* participate in digital contact tracing. Whether such an expectation is *unjust* arguably depends on the particular app, epidemiological background assumptions, substantive normative commitments, and broader societal negotiations (Braun and Hummel 2020). It is notable in any case that burdens of proof can shift in these ways, and care should be taken to reflect on what constitutes undue levels of intrusiveness in connection with digital contact tracing.

The fine line between incentivizing and disciplining becomes apparent in the case of corporate wellness programs and their increasing reliance on continuous tracking and big data analytics (Ajunwa et al. 2016). These programs are framed as benevolent towards the participating employee. They are also designed to generate benefits for employers and program service providers who process entrusted employee data. On the one hand, care is needed to ensure that incentivization of participation does not indirectly penalize non-participants who decide to refrain from sharing health data with their employer. On the other hand, for those who participate, tacit imperatives might be introduced and violations might receive sanctions, e.g., through employment discrimination. Especially in health systems where employers carry parts of the healthcare costs of employees, “there is the temptation for the thrifty employer to deputize wellness programs as surveillance systems that would root out ‘costly’ employees” (Ajunwa et al. 2016, 478). The interests of the employer and service provider might in fact take precedence over the interests of the employee. Again, big data analytics make these systems as effective as it gets, and if deployed uncarefully result in individuals being denied certain basic goods for partaking in society.

A further issue besides pressures to track data concerns pressures to *improve* the data being tracked. Pervasive health data tracking can urge individuals to comply with logics of normalization and optimization, and discredit ways of life that result in deviations from biostatistical benchmarks (Braun and Dabrock 2016), for example when insurance bonuses are tied to favorable or improved vitals and a corresponding sanctioning of undocumented or less favorable health data. One particular difficulty with such setups in a health system is that they rest too easily on *presumed* equity, e.g., with regards to available opportunities to enjoy good health. This presumption neglects, firstly, the multifactorial etiologies mentioned above, and secondly, the fact that certain biomedical and social determinants of health are beyond the reach of individual voluntariness and will-formation. It would threaten to result in misguided applications of notions of fairness, performance, and achievement if individuals are held to standards whose fulfillment they cannot affect. Datafication, the sharing of vitals, and incentivization of either favorable vitals or the mere act of tracking can invite overstatements and false impressions of individual responsibility for health (German Ethics Council 2017, 224–25). These points do not deflate the value of data gathering per se. But they prompt us to honor the ideal of voluntariness when shaping these practices, and to address potential disadvantages for individuals who decide to refrain from participating in such activities.

The three kinds of injustices just described can arise through and be reinforced by data-driven medicine. Drawing the foregoing results together, data-driven medicine rests upon desiderata which pull in conflicting directions: the need for data from a pragmatic standpoint and the perspective of the *good* versus drawbacks in the sphere of the *right*. These challenges might have been always implicit to health data sharing and analysis, but they are amplified drastically by big data, the potential to reidentify data subjects even if data were anonymized (Rocher et al. 2019), and the difficulty to tell which data have or could have implications about health.

## Key elements for aligning the good and the right in data-driven medicine

Data, and especially personal health data in an environment shaped by a merger of bio- and information technology, can cause, perpetuate, and amplify injustices. Their invasiveness, their various effects on decision-making, and the range of potentially affected people complicate the design of sound and effective governance frameworks. This, however, is certainly not a reason to refrain from advancing data-driven methods in biomedical research and care altogether. First, we have just argued that such methods yield potentials from the perspective of the *good.* Second, while we have pointed out that seeking to realize these potentials can ultimately compromise the *right*, we should not lose sight of the fact that under the right conditions, these methods can also *strengthen* the *right*. For example, it is certainly possible that AI-driven medicine provides opportunities to *enhance* rather than to inhibit health equity, e.g., by facilitating health coverage in low-resource settings (World Health Organization 2018). The general question thus becomes not whether, but *how* we can develop and deploy data-driven biomedical innovation and harness these tools to facilitate solidarity and to promote the common good, while at the same mitigating drawbacks for individual rights as far as possible.

One first step is the *controllability* of data flows. Again, empirical evidence can provide cues. In their systematic review of published qualitative studies on attitudes towards genomic data sharing, Shabani et al. (2014) conclude that besides the desire to help others and to contribute to the greater good, some individuals hold expectations about the controllability of shared genomic information. Some of them even portray controllability as an inherent right that individuals take themselves to possess independently of any consequences that arise from the processing of genomic information, and a condition that would signify respect and honor their involvement. Correspondingly, shortcomings in the controllability of information flows are perceived as a barrier to sharing. Some individuals thus demand control mechanisms that are fine-grained enough to determine what kinds of purposes and studies their data serve, but lenient enough to avoid burdensome requests on each and every request of access and use. Importantly, some maintain that blanket consent requirements would fall short of such kind and degree of control. This is significant insofar as some authors take it to be obvious that broad consent procedures will be necessary for big-data-driven research. They thus focus on evaluating and optimizing forms and wordings to ensure that relevant information on the breadth of consent and the framework for usage is being conveyed (Strech 2018). In contrast, the foregoing suggests that the ideal of controllability calls for more.

We propose to understand the *controllability* of data flows as encompassing the availability of means to know which kind of person-related inferences can be drawn on the basis of one’s data in order to consider possible impacts on the individual modes of freedom. On this basis, controllability then further involves an entitlement to have means to protect, to share, but also to retract information (Hummel et al. 2019). As one specific example, Wachter and Mittelstadt (2019) demand a *right to reasonable inferences* which motivates an entitlement of data subjects to *ex-ante* justification concerning the *reasonableness* of data-driven inferences to which they are subjected, e.g., the kind of data and their source, the purpose of data processing, and the statistical reliability about the inference. It also encompasses a right to challenge inferences if they seem unreasonable along these dimensions. However, little has been said on what such a right would mean in medical contexts. We are convinced that controllability would imply the possibility to make one’s data available for data-driven applications, but also to *forego* such technologies, for example when the individual cannot rule out unwanted disclosure of personal and intimate information, or expects disproportionate increases in vulnerability from sharing her data. For these reasons, the ideal of controllability is in tension with attempts to *broaden* the scope of consent that, although still relying on voluntariness, effectively result in losses of control over one’s data. Instead, it motivates *dynamic* consent procedures which allow individuals to exercise continuous control. This involves real-time management of their data, determining which kinds of information become accessible to whom, choosing research projects and tiers that may process data, and defining certain contexts of use that are off-limits. Moreover, it would require consistent technical and legal implementation of the concept of data portability and deletion by enabling individuals to withdraw data entirely once they prefer to re-establish privacy.

Immediately related to the foregoing, technical and system-level *structures* would have to be in place as enabling conditions for controllability. This includes sufficient degrees of data interoperability in order to be in a position to submit and transfer data from, e.g., electronic health records and direct-to-consumer genetic testing to data-intensive research projects. Only then will it be possible to compare, translate, conjoin, exchange, and connect data at a reasonable scale. Moreover, programmatic interfaces would have to be aligned, harnessed, and made accessible in user-friendly ways to ensure the feasibility of organizing, filtering, and directing data as envisioned in dynamic consent.

One technological infrastructure which is frequently discussed as a possible avenue to pursue the ideal of controllability is the blockchain technology. Data networks normally require a specific form of gatekeeping: typically, an organization or system maintains the database, governs access, guarantees for the integrity of the network, and hence is the addressee of trust from the participating data subjects. With a technology like blockchain, the network *itself* and the logs of past process and transactions in the blockchain could constitute a form of *technological* custodianship. The idea of such a technological custodianship would be that there is no third party who mediates between data donor and processor, that the technological design is receptive to the preferences and expectations of the donor, and that it guarantees that data are handled accordingly throughout the process of data collection and processing. Its decentral and open architecture promises to be less prone to power asymmetries that tend to arise in the platform economy. Immutable audit logs and access as well as verification mechanisms based on cryptography might make it possible to build networks in which users can share information, have it protected, and retain control of who has and should have access. By offering an alluring combination of transparency, secrecy, control, and decentralization, blockchain could offer structural enablers for informational self-determination. Several approaches are currently emerging on how to apply blockchain technology to governing access to health data (Azaria et al. 2016; Kuo et al. 2017; Xia et al. 2017; Porsdam Mann et al. 2020).

A variety of blockchain technologies try to seize these potentials (Süssenguth et al. 2018). Interestingly, many of them still seem to rest upon institutional frames that render the network functional in the first place. Non-public blockchain networks need gatekeepers, permissioned blockchains have operators, and all of them require designers and engineers who define, maintain, and refine rules and protocols. Decision-making processes on the governance of the network can remain opaque and be affected by mere power rather than genuine deliberation. Blockchain networks, too, have seen market concentrations that are somewhat at odds with the emancipatory and subversive spirit that drove their emergence (De Filippi 2017). Not always are gatekeepers straightforwardly identifiable and localizable in institutions inside and outside the network. For non-experts, it is not obvious how the network realizes custodianship, and who the addressee of expectations and claims could be. The idea that one can trust in the network itself is compromised if it becomes apparent that external agents and institutions exercise oversight and confer credibility.

Our claim is not that the controllability of one’s health data stands and falls with the opportunities and implementations of blockchain technology. The blockchain illustrates opportunities, challenges, and risks that other putative enabling technologies of controllability need to navigate, too. It raises questions on how we can make credible the idea that users can steer the flow of their information, and which conditions foster the trust that sets such systems in motion.

This is compatible with exploring other technological designs and approaches. For example, the promise of *differential privacy* is to provide algorithms and IT infrastructures that make coarse-grained data available while keeping information on individuals private: “You will not be affected, adversely or otherwise, by allowing your data to be used in any study or analysis […]. Differential privacy addresses the paradox of learning nothing about an individual while learning useful information about a population” (Dwork and Roth 2013). Still, while there is no doubt that such architectures are beneficial, they are plausibly seen as complements rather than surrogates for *dynamic* control mechanisms. Even if an algorithmic output is *differentially private* by virtue of revealing nothing about the individuals whose data informed the output, they and others could still be affected along the same lines that motivate the kind of group privacy rights mentioned above. The ideal of informational self-determination and *dynamic* control mechanisms would be that data subjects are in a position to affect in real-time the extent to which their data is available both for the process of ad hoc grouping (Mittelstadt 2017) and for classifying the individual as sharing relevant features with one ad hoc group or other.

A second step beyond the individual controllability of data flows is to broaden the scope of the proposed ethics of data-driven medicine beyond the individual level. Through a paradigm shift in policy and governance from input- towards *output-orientation* (Dabrock 2018), attention should be devoted to the downstream effects of data-intensive tools on the freedom, opportunities, and conditions for implementing life plans and decisions. Traditional data-protection is input-oriented by focusing on conditions for feeding data into processing, such as informed consent, data minimization, and purpose limitation. Reflecting on such conditions has its place. The power, pervasiveness, and coordinative roles of data-intensive tools also suggest that there is a need for more, and that Mittelstadt et al. (2016) are right to demand that the *implementation* and *configuration* of algorithms must be taken into consideration. These tools steer and mediate a variety of processes across social spheres, and thus affect the circumstances in which individual decision-making takes place. As the example of the *healthy ill* demonstrated, unjust gaps in service coverage can arise or become apparent *after* individuals have autonomously consented to having their risk profiles uncovered. Where the pursuit of individual life plans, their integration into communal and societal structures, and the de facto access to participation, deliberative spaces, and basic goods are compromised—in this case by the subjective state of *illness* (as opposed to acute, socially recognized and biomedically determinable *disease*)—output-orientation kicks in and adjustments to alleviate such effects become necessary. Or consider again the examples of health data tracking and digital contact tracing. Surely input-oriented conditions can apply, and entitle subjects to refrain from sharing health data with employers and their service providers, or from using a tracing app. But the very existence and introduction of these programs already stacks the deck a certain way, and at the output stage, tacit disciplining structures as well as shifts in burdens of proof can result and frame choices, refusals, and expectations related to informational self-determination—even if autonomy is superficially left intact at the input stage.

Our suggestions are driven by the conviction that a responsible ethics of data-driven medicine cannot be restricted to protecting individuals and their data from being processed by and be subject to algorithmic tools. We cannot limit ourselves to driving wedges between algorithms, individuals, and the lifeworld. Apart from potentials to utility and beneficence that should be harnessed, the presence of motivations to enact solidarity by contributing to research and the common good gives strong reasons to enable individuals to actively feed data into algorithms. Thus, not restraint, but a governance mix towards channeling information flows, enabling their controllability, and responding to downstream effects is needed.

If implemented with care, these conditions could harmonize the significance of contributing data from the perspective of the *good* with effects of such contributions that matter from the perspective of the *right*. Individuals will in principle be in a position to share their data, to contribute them to data-driven tools, and thereby to pursue motivations related to solidarity or self-interest. Where data or the consideration of data are skewed, output-orientation kicks in and, for example in the dermatology case, mandates equitable consideration of data from all populations. Controllability means that individuals continuously remain in a position to retract and shield data from contexts of use. Once again, output-orientation requires that no disadvantages, e.g., with regards to opportunities and access to basic goods, materialize as a consequence of data-restrictive choices. This includes addressing and compensating for vulnerabilities such as disease risk profiles uncovered by data-driven medicine. Against the backdrop of suitable IT structures and output-orientation in governance, controllability can serve as a means to mediate between solidarity and justice.

## Conclusion

With a sectoral focus on sharing *health* data in the context of data-driven medicine and clinical big data applications, two contributions to an ethics of data-driven medicine have been offered. First, we have pointed at two different pursuits that we might subsume under the heading of ‘ethics’, and which extant theories of the ethics of data-driven medicine do not yet distinguish: an account of how we can advance the *good* versus a theory of what it takes to secure the *right*. Second, we have argued that with regards to data-driven medicine, making health data available is to be welcomed from the perspective of the *good*. It is a necessary condition for realizing the potentials of data-driven medicine, and for allowing individuals to enact solidarity and to contribute their data to research. However, the challenge we have identified in this context is that such endeavors can backfire. Attempts to promote the *good* through making health data accessible can culminate in constraints in the sphere of the *right*. Specifically, we considered the examples of inequitable consideration of data from different sources and populations, shortcomings in distributing access to data-driven medicine and addressing risk profiles uncovered by its predictive methods, and unjust introductions and disciplining effects of continuous tracking of vitals.

Our positive suggestion is that data sharing practices, whether driven by self-interest or in order to enact solidarity, should be made possible in ways that are mindful of potential injustices. Specifically, we proposed that the controllability of data flows, infrastructures conducive to controllability, and a shift in focus from input- to output-orientation in governance are key for preventing and mitigating injustices. These conditions put individuals in a position to share data, to retract them if necessary or desired, and to rely on the mitigation of unjust downstream effects of data-intensive tools.

A full-fledged ethics of data-driven medicine needs to consider the range of attitudes, concerns, and motivations of individuals who contribute to these technologies and are affected by them. Foundational norms of justice set the stage for offering and receiving gifts of solidarity that stand the chance of generating and sustaining community. On these foundations, tools such as COVID-19 tracing apps might in the best case promote the *good* without compromising the *right*.

## Data Availability

Not applicable.
